# Vibration direction sensitivity of the cochlea with bone conduction stimulation in guinea pigs

**DOI:** 10.1038/s41598-021-82268-3

**Published:** 2021-02-03

**Authors:** Mingduo Zhao, Anders Fridberger, Stefan Stenfelt

**Affiliations:** Department of Biomedical and Clinical Sciences, Linköping University, 58185 Linköping, Sweden

**Keywords:** Neurology, Inner ear, Auditory system, Cochlea

## Abstract

Sound and vibrations that cause the skull bone to vibrate can be heard as ordinary sounds and this is termed hearing by bone conduction (BC). Not all mechanisms that causes a skull vibration to result in BC hearing are known, and one such unknown is how the direction of the vibration influences BC hearing. This direction sensitivity was investigated by providing BC stimulation in five different directions at the vertex of the guinea pig skull. The hearing thresholds for BC stimulation was obtained in the frequency range of 2 to 20 kHz by measurements of compound action potential. During the stimulation by BC, the vibration of the cochlear promontory was measured with a three-dimensional laser Doppler vibrometer resulting in a set of unique three-dimensional velocity magnitude combinations for each threshold estimation. The sets of three-dimensional velocity magnitude at threshold were used to investigate nine different predictors of BC hearing based on cochlear promontory velocity magnitudes, six single direction (x, y and z directions in isolation, the normal to the stapes footplate, the oval to round window direction, and the cochlear base to apex direction), one linear combination of the three dimension velocity magnitudes, one square-rooted sum of the squared velocity magnitudes, and one sum of the weighted three dimensional velocity magnitudes based on a restricted minimum square error (MSE) estimation. The MSE gave the best predictions of the hearing threshold based on the cochlear promontory velocity magnitudes while using only a single direction gave the worst predictions of the hearing thresholds overall. According to the MSE estimation, at frequencies up to 8 kHz the vibration direction between the right and left side gave the greatest contribution to BC hearing in the guinea pig while at the highest frequencies measured, 16 and 20 kHz, the anteroposterior direction of the guinea pig head gave the greatest contribution.

## Introduction

The two main pathways of hearing, air conduction (AC) and bone conduction (BC), have been studied extensively during the last two centuries. When vibrations are transmitted in the skull bone, the hearing sensations by BC are perceived the same as with normal AC^1–3^. Early on, sound transmission by BC was assumed similar to the waves on a water surface, which means that the vibration is only in one direction, forming a transversal wave, perpendicular to the surface^4^. Later it was understood that when the wave propagated across the cranial vault, it comprised both longitudinal and transverse components and at low frequencies, the human skull behaves like a rigid body^5^. Since then, multiple studies have been conducted to investigate the mode of vibration in the skull during BC stimulation^6–11^.These studies have confirmed that the skull vibration is complex and frequency dependent during BC stimulation. Such complex motion prevents an easy transformation of the skull bone vibration to a sound perception. Consequently, at present, it is not known how different types of wave modes and vibration directions influence hearing by BC stimulation.

For AC hearing, sound vibration propagates in the air, is collected by the external ear (pinnae) and is transmitted to the middle ear ossicular chain (malleus, incus, and stapes) via the eardrum and finally excites the fluids in the inner ear (cochlea) causing a motion of the basilar membrane to generate a hearing sensation^12–14^. Hearing by BC involves several contributing pathways^1,15,16^. The BC stimulation can be either directly to the skull bone or on the skin over the skull, and transmitted as vibrations of the skull to the inner ear, through the ear canal and middle ear, or through the cerebrospinal fluid (CSF). Irrespective of pathway for the vibratory energy^17^, all integrate in the inner ear and cause a motion of the basilar membrane generating a hearing sensation^1,18^.

For AC stimulation, the motion of the stapes is often used as an approximation of the hearing stimulation^19,20^. For BC stimulation, neither the middle ear ossicles motion nor the round window motion can be used to assess the hearing excitation ^20,21^. Most studies that use vibration as a means to assess the BC excitation use the vibration magnitude of the cochlear promontory as an estimate of the hearing stimulation. The rationale is that the vibration magnitude of the bone around the inner ear seem to be a dominant pathway for BC hearing in the human^15^ and the cochlear promontory vibration magnitude correlates with perceived BC hearing^9^.

The most common estimate of BC perception based on cochlear promontory vibration is a one-dimensional motion, often obtained by a laser beam through the ear canal or a surgical opening in the mastoid behind the ear^5,7,10^. However, the bone around the cochlea vibrates in all three space dimensions, and it is currently not known for what direction or type of wave motion the inner ear is most sensitive. One approach to accommodate for this uncertainty is to use the square-root of the sum of the orthogonal vibration components squared^5^. The rationale for this estimate is that it represents the vibrational energy of the cochlear promontory and it is less affected by resonances or anti-resonances in the transmission pathway that can appear in a single direction. A third proposed estimate of the BC perception is to compute the main vibrational direction and use this maximum as a proxy of the BC sound^22^. One advantage of the latter is that it contains the phase information of the vibration which is important for understanding the mode of vibration.

There are indications that the vibration direction of the skull bone influences the BC perception^23–26^. Bárány^23^ argued that the shape of the middle ear bones evolved to not only maximize hearing by AC, but also to minimize the hearing of one’s own voice and other internally generated sounds by the BC pathway. Von Békésy^24^ was of the same opinion as Barany, and further indicated that the ossicles might lie along an axis that minimizes their vibrations resulting from phonation. Von Békésy^24^ claimed that the vocal cords produce maximum vibrations along the vertical direction, but that their vibrations in the direction of the ear canal axes were smaller. This hypothesis was never experimentally verified. In a study on the middle ear ossicles vibrations during BC excitation in human temporal bone specimens, it was found that the effective vibration of the stapes was within 5 dB regardless of the vibration direction, thus disproving the hypothesis by Bárány and von Békésy^27^. Moreover, both experimental and modeling studies have indicated that the influence from the middle ear is some 10 dB below other contributors for BC hearing in the human^15,28,29^.

Finite element models (FEM) of the human head and ear have been used to investigate the influence of the vibration direction for BC sound. In a whole human head FEM for auditory BC research^25^, calculations of the directional sensitivity of the cochlear vibration magnitudes showed small differences between the 3 orthogonal direction, where the difference between the maximum and minimum sensitivity was approximately 5 dB. In another study of a FEM of the inner ear, the response for vibration approximately in line with the axis of the oval and round window was greater than the responses from vibrations in other directions^26^. One limitation of that study was that only inertial effects was included while both inertial and compressional motion is important for BC sound perception^18^.

Consequently, neither experimental nor computational studies have resolved the issue of directional sensitivity for BC sound. The aim of the current study is to investigate the BC hearing sensitivity to the vibration magnitudes in three orthogonal directions of the cochlear bone and relate those directions to the anatomy of the inner ear. This is executed in guinea pigs where we recently developed a method for relating the BC hearing threshold to cochlear vibrations^30^. The BC stimulation is applied to the guinea pig head in different directions, and the resulting three-dimensional velocity magnitudes at the cochlear promontory are obtained as well as the hearing threshold. By computing the cochlear velocity magnitudes in three dimensions at threshold for several stimulation directions, estimates of the directional sensitivity for BC vibration of the cochlea are obtained in the guinea pig.

## Results

**Figure 1:**
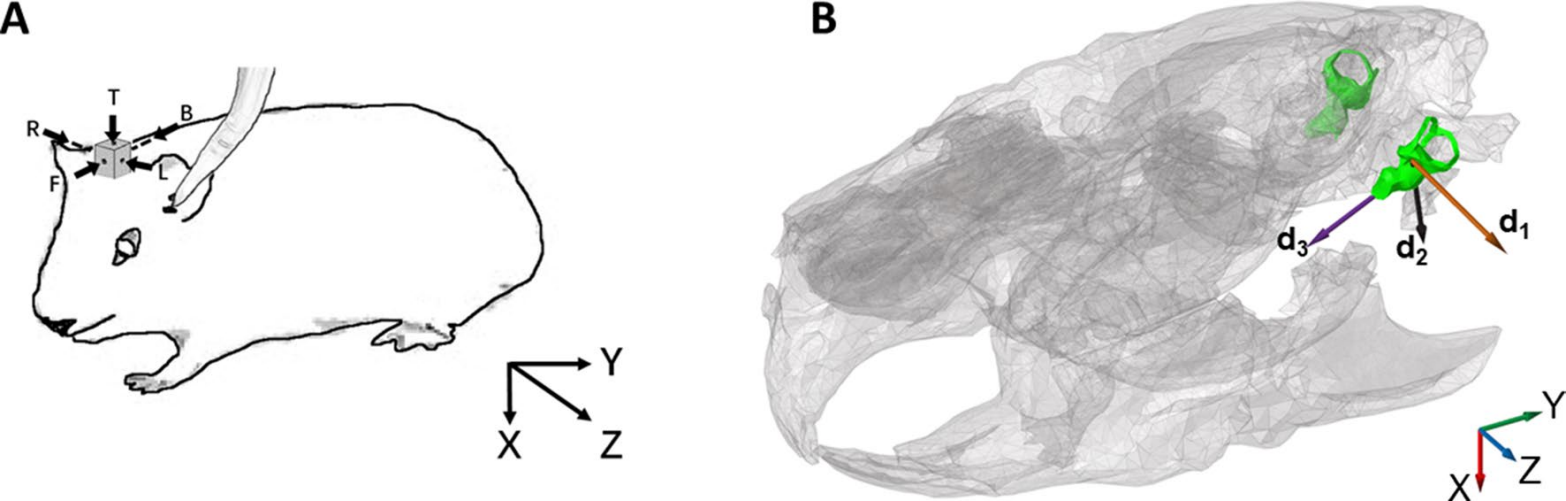
The guinea pig. (**A**) The BC transducer is attached to the brass cuboid and the tube for AC stimulation is placed at the ear canal opening. The coordinate system indicates the directions used for the vibration measurement with the laser Doppler vibrometer where x-direction is ventral, y-direction is posterior, and z-direction is left. (**B**) The skull bone of a guinea pig with the inner ears shown in green. The bone around the left cochlea has been removed for visibility purpose. The anatomical references for vectors ***d***_***1***_, ***d***_***2***_ and ***d***_***3***_ are given in the text.

The hearing thresholds in guinea pigs with AC stimulation and a detailed analysis of the BC thresholds with the BC transducer mounted on the top stimulation position can be found in Zhao et al.^30^. Here, the focus is on the threshold predictions based on the 3D vibration of the cochlear promontory. The stimulation directions were aligned with the x, y and z directions in overall coordinate system (Fig. 1A). CT scans of one guinea pig head were obtained and segmented to relate the anatomy of the inner ears to the overall coordinate system (Fig. 1B). Three vectors based on the left side cochlear anatomy were identified and visualized in Fig. 1B where vector ***d***_***1***_ is the normal direction of the stapes footplate, ***d***_***2***_ is the direction from the center of the oval window to the center of the round window, and ***d***_***3***_ is aligned with the center of the cochlea going from the base towards the apex. These three vectors are relatively well aligned with the overall coordinate system and are nearly orthogonal to each other. The relation between the coordinate system and the three vectors are shown in Table 1 indicating that the direction normal to the stapes footplate (***d***_***1***_) is mainly in the z direction, the oval to round window direction (***d***_***2***_) is mainly in the x direction, and the base to apex of the cochlea (***d***_***3***_) is mainly in the reversed y direction.

**Table 1:** The directions of the cochlear vectors in Fig. 1 in relation to the overall coordinate system. *e*_*x*_, *e*_*y*_, and *e*_*z*_ are the base vectors for the x, y and z directions, respectively.

Direction	Relation	Eq. No
Stapes footplate normal	${d}_{1}=0.50{e}_{x}+0.35{e}_{y}+0.79{e}_{z}$	(1.1)
Oval window to round window	${d}_{2}=0.92{e}_{x}+0.25{e}_{y}-0.28{e}_{z}$	(1.2)
Cochlear base to apex	${d}_{3}=0.38{e}_{x}-0.92{e}_{y}+0.05{e}_{z}$	(1.3)

**Figure 2:**
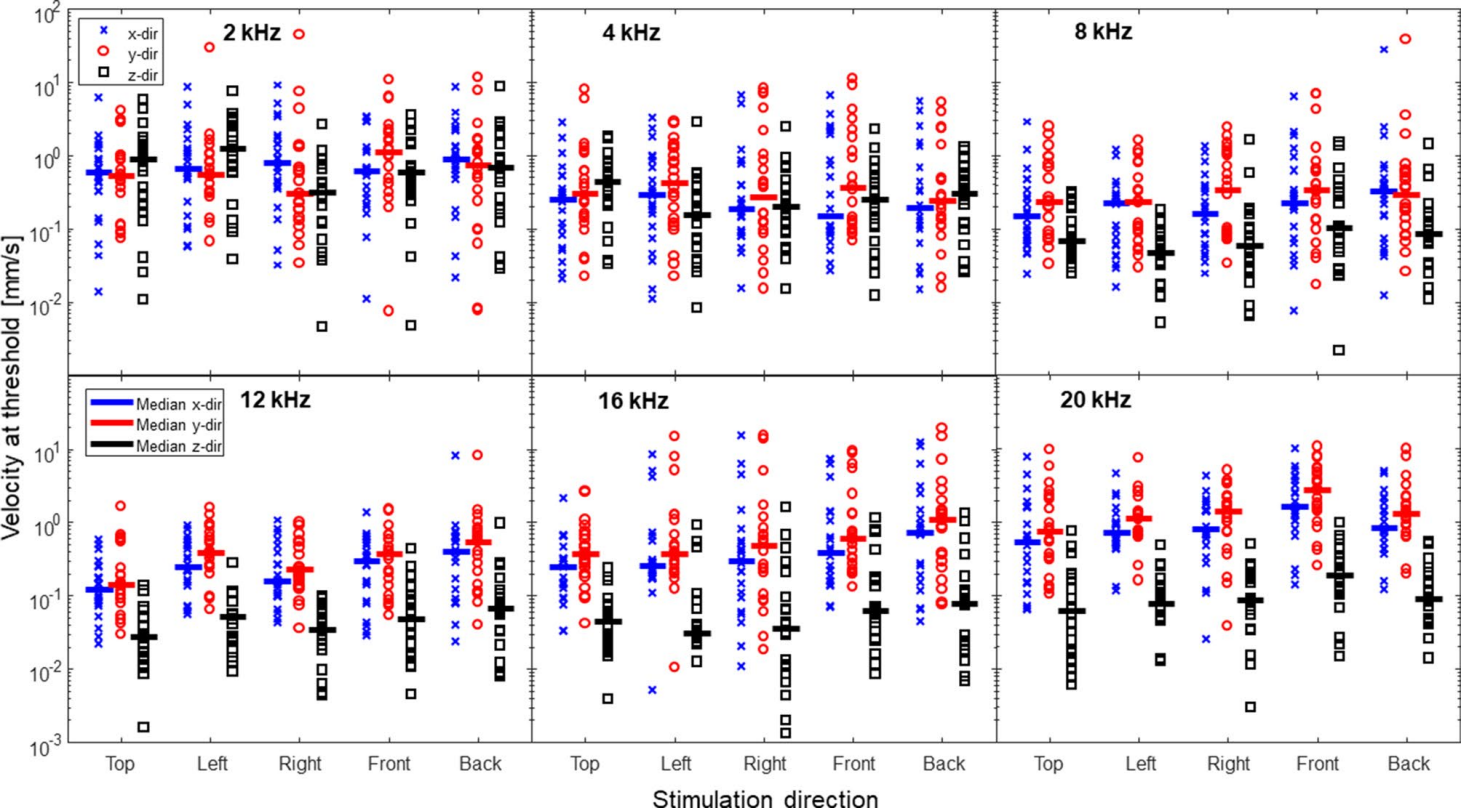
The individual vibration magnitudes at threshold in the three orthogonal directions when the stimulation is in the five different directions. Each marker indicates an individual guinea pigs velocity magnitude at threshold (CAP = 0 V) and the solid lines indicate the median for each frequency and response direction.

Figure 2 displays the individual velocity magnitudes at threshold in the three orthogonal directions when the stimulations are in the five different directions. The figure illustrates a variability in velocity magnitude at threshold where most results are within a 30–40 dB range. The velocity magnitude at threshold data in Fig. 2 also show that the velocity magnitudes are similar in the three directions at the frequencies between 2 and 8 kHz, while the z direction has significantly lower velocity magnitudes than the x and y directions at 12–20 kHz.

**Figure 3:**
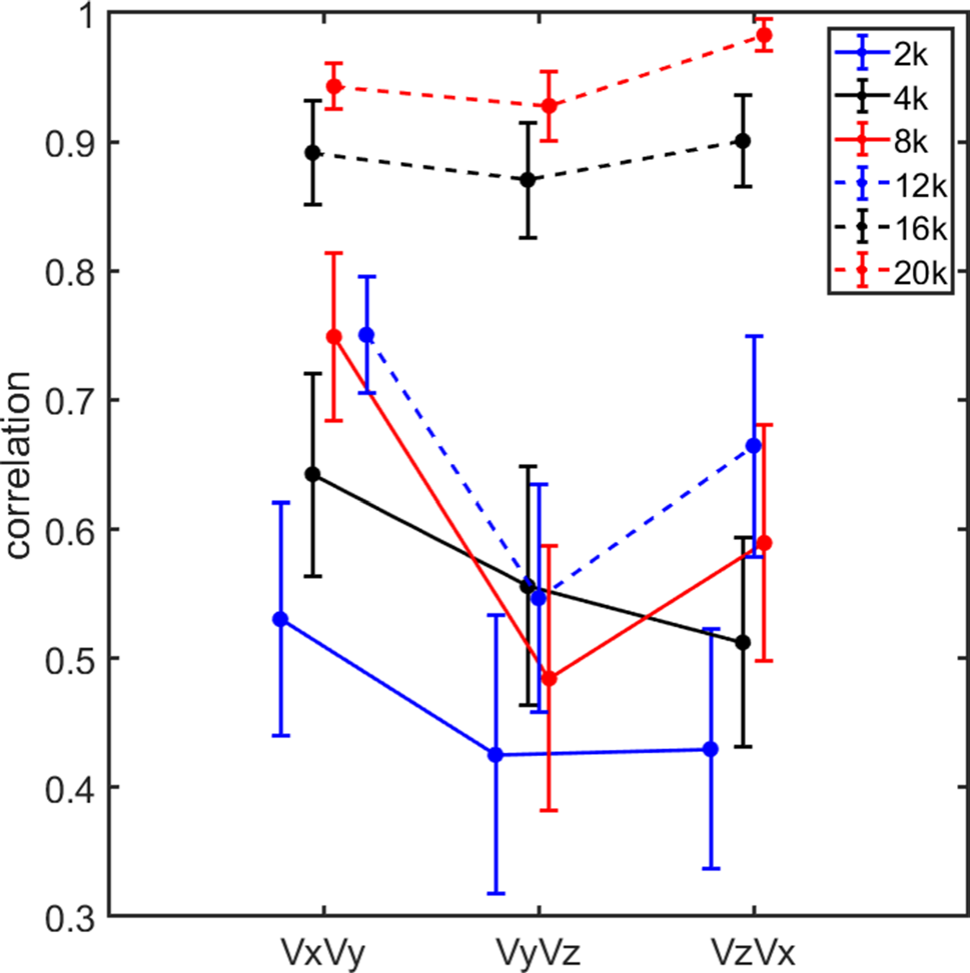
The average correlations between velocities at threshold. The correlations is obtained for all guinea pigs when the stimulations were at the five different positions. The correlations between the three response direction combinations are shown for each frequency and the error bars indicates the standard error of the mean.

One prerequisite for the analysis in the current study is that the relative vibration levels in the three directions at the cochlear promontory differ between stimulation directions. The more different the x, y, and z direction velocity magnitudes are at threshold for the five stimulation directions, the better the specificity of the cochlear bone vibration directions’ influence on BC hearing can be determined. The similarity of vibration levels at threshold for the 5 stimulation directions was here evaluated by computing the correlation between the velocity magnitudes at threshold for the response directions pairwise, i.e. the correlation is computed as the velocity magnitude at threshold between x and y directions, x and z directions, and y and z directions for stimulations in the five directions. These correlations are averaged over all guinea pigs and displayed for the three response direction combinations and 6 frequencies in Fig. 3. The overall correlations increased with frequency where the lowest average correlation was 0.42 between the velocities in the y and z directions at 2 kHz while the highest average correlation was 0.98 between the velocities in the x and z directions at 20 kHz. Consequently, at low frequencies, the three-dimensional response velocity magnitudes at the cochlea differs when the stimulation is applied in different directions while at the highest frequencies, the three-dimensional velocity response magnitudes are almost independent of the stimulation direction. This means that the response velocity magnitude at the cochlear promontory depend on the stimulation direction more at low frequencies than at high frequencies. The relatively high overall correlations obtained between the velocities at threshold (0.42 to 0.98) are a result of front–back and left–right stimulations that are expected to produce the same spatial vibration magnitudes at the cochlear promontory.

**Figure 4:**
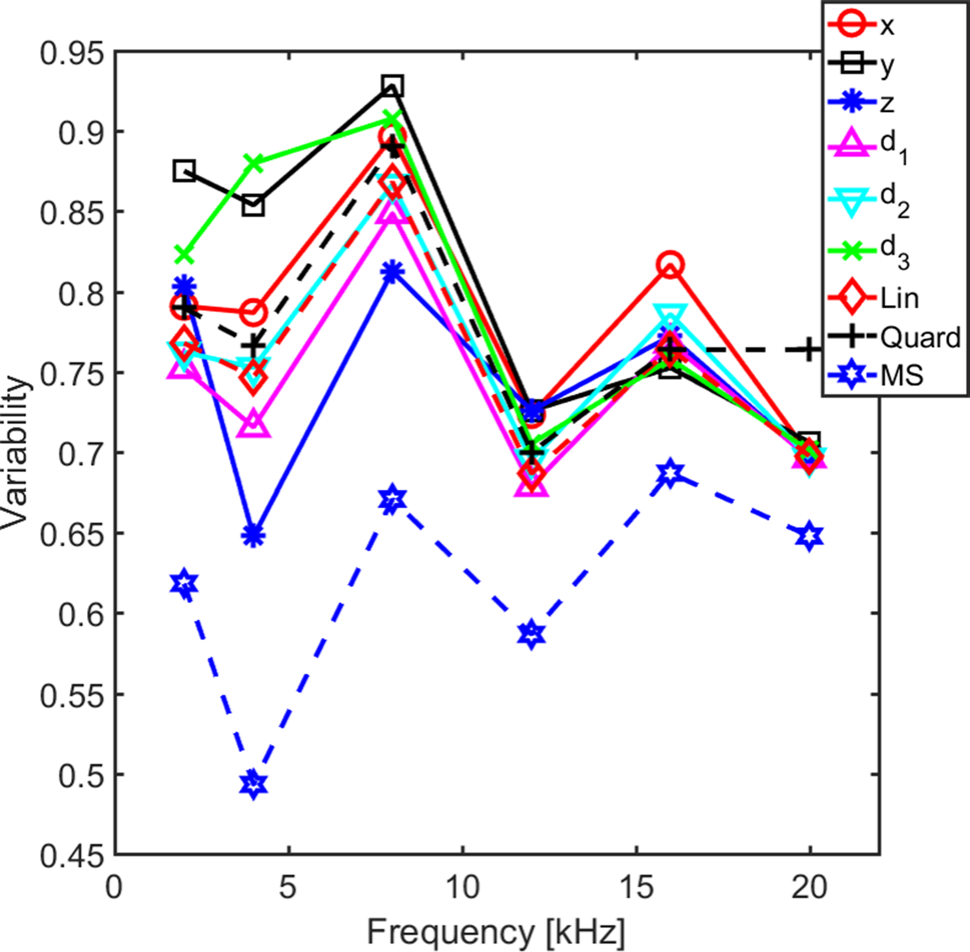
The variability of the six different threshold estimation methods. The variability is computed according to Eq. (2) for the nine different threshold estimations methods.

The threshold predictions based on the nine different combinations (Table 2) of the orthogonal velocity magnitudes at threshold were evaluated by two methods. The first method was a variability estimate of the predictions. This was a root-mean-square calculation over stimulation directions (*n* = 5) and number of animals (*n* = 22) according to the equation

2 $$var(f)= \sqrt{\frac{1}{{n}_{animal}}\sum_{i}\left(\frac{1}{{n}_{stimulation}}\sum_{k}{\left(\frac{{v}_{i,k}(f)-\stackrel{-}{{v}_{i}(f)}}{\stackrel{-}{{v}_{i}}(f)}\right)}^{2}\right),}$$

where *f* is the stimulation frequency, index *i* stands for the *i*th animal, index *k* stands for the *k*th stimulation direction, *v* is the combinations of velocity magnitudes according to the estimation function (Eqs. 4.1 to 4.9), and $\stackrel{-}{v}$ is the average velocity at threshold according to the estimation function. The lower the variability for the estimation function the better the function predicts the hearing threshold based on the combination of velocity magnitudes. The results from this variability computation for all nine different estimation combinations and frequencies are shown in Fig. 4. According to Fig. 4, the best threshold prediction is obtained when the three velocity magnitudes are combined according to the MSE method. The variability of the MSE method is 0.1 to 0.15 lower than the best predictor of the other methods. It is not surprising that the MSE method outperforms the other methods on the variability measure since the MSE method computes the three weighting coefficients (*a*, *b*, and *c*, Eq. 4.6) that minimizes a cost function that is very similar to the variability measure in Eq. (2).

**Table 2:** Names and equations for the nine velocity functions used in the current analysis. ^*^ indicates complex conjugate.

Name	Equation	Eq. No
x direction	$v=\left|{v}_{x}\right|$	(4.1)
y direction	$v=\left|{v}_{y}\right|$	(4.2)
z direction	$v=\left|{v}_{z}\right|$	(4.3)
Linear sum	$v=\left|{v}_{x}\right|+\left|{v}_{y}\right|+\left|{v}_{z}\right|$	(4.4)
RMS	$v=\sqrt{{{v}_{x}\bullet v}_{x}^{*}+{v}_{y}\bullet {v}_{y}^{*}+{{v}_{z}\bullet v}_{z}^{*}}$	(4.5)
MSE	$v=a\bullet \left|{v}_{x}\right|+b\bullet \left|{v}_{y}\right|+c\bullet \left|{v}_{z}\right|$	(4.6)
Stapes footplate normal (***d***_***1***_)	$v=0.50\bullet \left|{v}_{x}\right|+0.35\bullet \left|{v}_{y}\right|+0.79\bullet \left|{v}_{z}\right|$	(4.7)
Oval to round window (***d***_***2***_)	$v=0.92\bullet \left|{v}_{x}\right|+0.25\bullet \left|{v}_{y}\right|+0.28\bullet \left|{v}_{z}\right|$	(4.8)
Cochlear base to apex (***d***_***3***_)	$v=0.38\bullet \left|{v}_{x}\right|+0.92\bullet \left|{v}_{y}\right|+0.05\bullet \left|{v}_{z}\right|$	(4.9)

For the other eight combinations of the velocity magnitudes, the variability in Fig. 4 varies over the test frequencies. At the lowest frequency, 2 kHz, the velocity magnitude in the y direction alone gives the greatest variability while the ***d***_***1***_ (stapes normal) direction gives the lowest variability of the eight, a difference of approximately 0.13. At 4 and 8 kHz, the z direction alone is the best predictor while the y and the ***d***_***3***_ direction alone is the worst predictor. At the three highest frequencies, the variability is more similar for the eight combinations and there is no clear trend. One interesting finding in the variability analysis is that linear summation of the velocity magnitudes (Eq. 4.4) result in a slightly lower variability than the quadratic summation (Eq. 4.5) that has often been used as a predictor of hearing stimulation based on vibrations. Another observation is that at frequencies up to 8 kHz there are directions that produce lower variability than adding the velocity magnitudes in all three directions, either linearly or as the root of quadratic summation, but at the higher frequencies, no direction seem to give a clear advantage.

**Figure 5:**
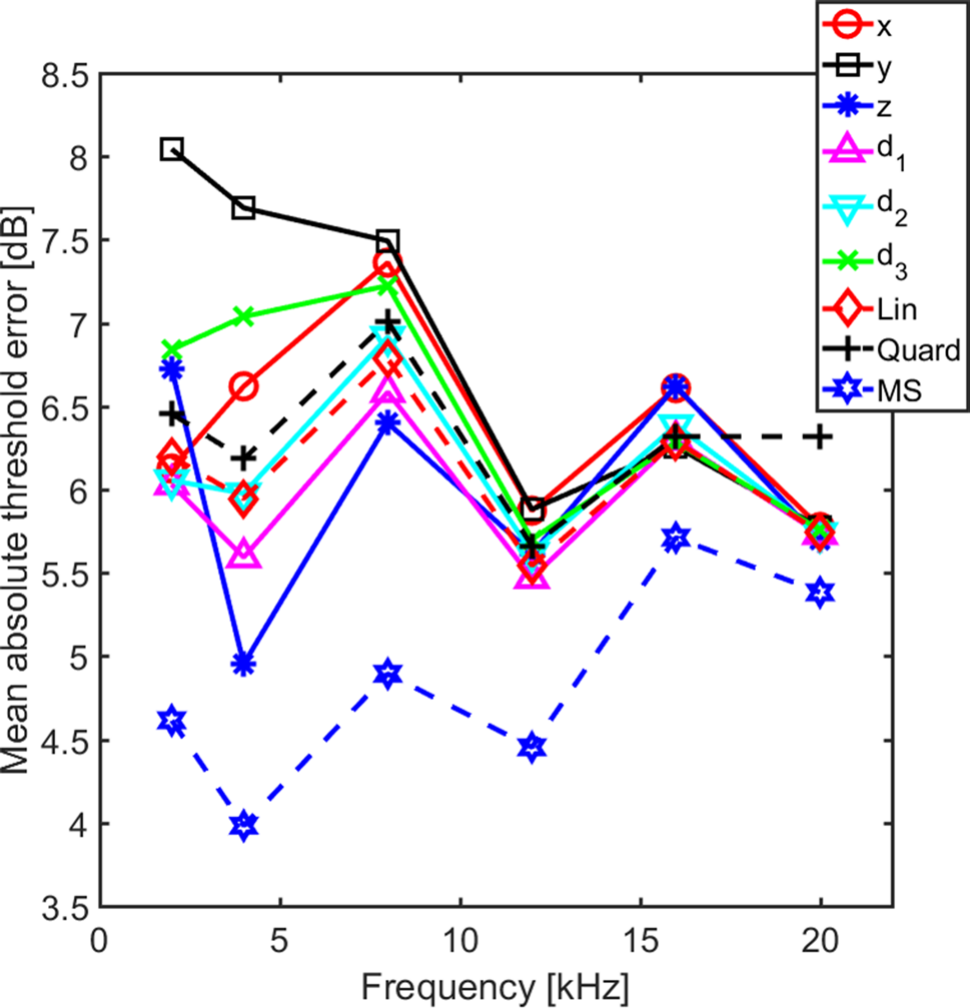
The average of the absolute threshold error for the nine different estimation methods.

Even if the variability analysis in Fig. 4 provides an estimate of the different velocity magnitude combinations’ ability to predict the hearing threshold, it is difficult to interpret the results in terms of a threshold magnitude error. Therefore, an analysis of the absolute threshold error is conducted. For each animal and frequency, the absolute difference in decibels between the predicted and measured hearing threshold is computed. The average absolute threshold error for the six different estimation methods are presented in Fig. 5. As with the variability analysis, the MSE method gives the lowest average errors, here between 4 and 5 dB at frequencies up to 12 kHz and around 5.5 dB at the two highest frequencies tested. Consequently, using a MSE method to predict the influence from the velocity magnitudes in three orthogonal directions on the hearing threshold in guinea pigs gives on average an error of 4 to 6 dB in the frequency range between 2 and 20 kHz.

The other eight combinations of the three velocity magnitudes result in 5 to 8 dB average threshold estimation errors. The worst prediction was obtained by only using the y direction velocity magnitude at 2 kHz while at frequencies at and above 8 kHz, all estimations except the MSE method gave a mean absolute threshold error that fell within 0.5 dB of each other. Similar to the variance analysis in Fig. 4, a single direction could produce both the best and worst predictions of the threshold while the summation of the velocity magnitudes from all three directions resulted in predictions the fell between the extremes. Also, the quadratic summation gave slightly worse estimates than adding the three velocity magnitudes linearly.

The differences in average absolute threshold error in Fig. 5 were relatively small, and to investigate if the differences were significant, a repeated measures ANOVA was computed with the method and frequency as within subject factors. When analyzing the ANOVA, the Mauchlys test of sphericity was significant for both the method [χ^2^(35) = 285.44, p < 0.001] and frequency [χ^2^(14) = 31.73, p = 0.005], and the degrees of freedom was adjusted according to Greenhouse–Geisser. According to the adjusted ANOVA, the method was significant [F(2.39,50.19) = 18.42, p < 0.001], frequency was not significant while the interaction between method and frequency was significant [F(5.73,120.45) = 2.29, p = 0.042]. This indicates that there is a significant difference in the absolute threshold errors between the methods and that the absolute threshold errors at the tested frequencies differ between the methods. When the 95% confidence interval for the methods were analyzed, the MSE method was significantly better (lower threshold errors) than all other methods, the y direction results were significantly worse than all but the x and z direction results, and the ***d***_***3***_ direction results were significantly worse than the linear summation and ***d***_***1***_ direction results . All other 95% confidence intervals overlapped.

The MSE algorithm estimates one weight for each direction and frequency. The estimation of the three coefficients, *a*, *b*, and *c*, as weights for the three orthogonal velocities that resulted in the best fit for estimating the vibration at hearing threshold, can be used as an estimation of the relative importance of the motion in the three directions. The computation of the hearing threshold based on the three velocities for the MSE method is according to Eq. 4.6 where $a\cdot \left|{v}_{x}\right|$ is the contribution from the velocity magnitude in the x direction on the hearing (here termed *C*_*x*_), and $b\cdot \left|{v}_{y}\right|$ and $c\cdot \left|{v}_{z}\right|$ are the y and z directions contributions (*C*_*y*_ and *C*_*z*_), respectively. The relative contributions from the three directions was here analysed by comparison of *C*_*x*_, *C*_*y*_, and *C*_*z*_. To facilitate comparison between animals, the contributors were normalized for each animal according to

3 $${S}_{i}=\frac{{C}_{i}}{\sqrt{{C}_{x}^{2}+{C}_{y}^{2}+{C}_{z}^{2}}}$$

where *i* is either *x*, *y* or *z*.

**Figure 6:**
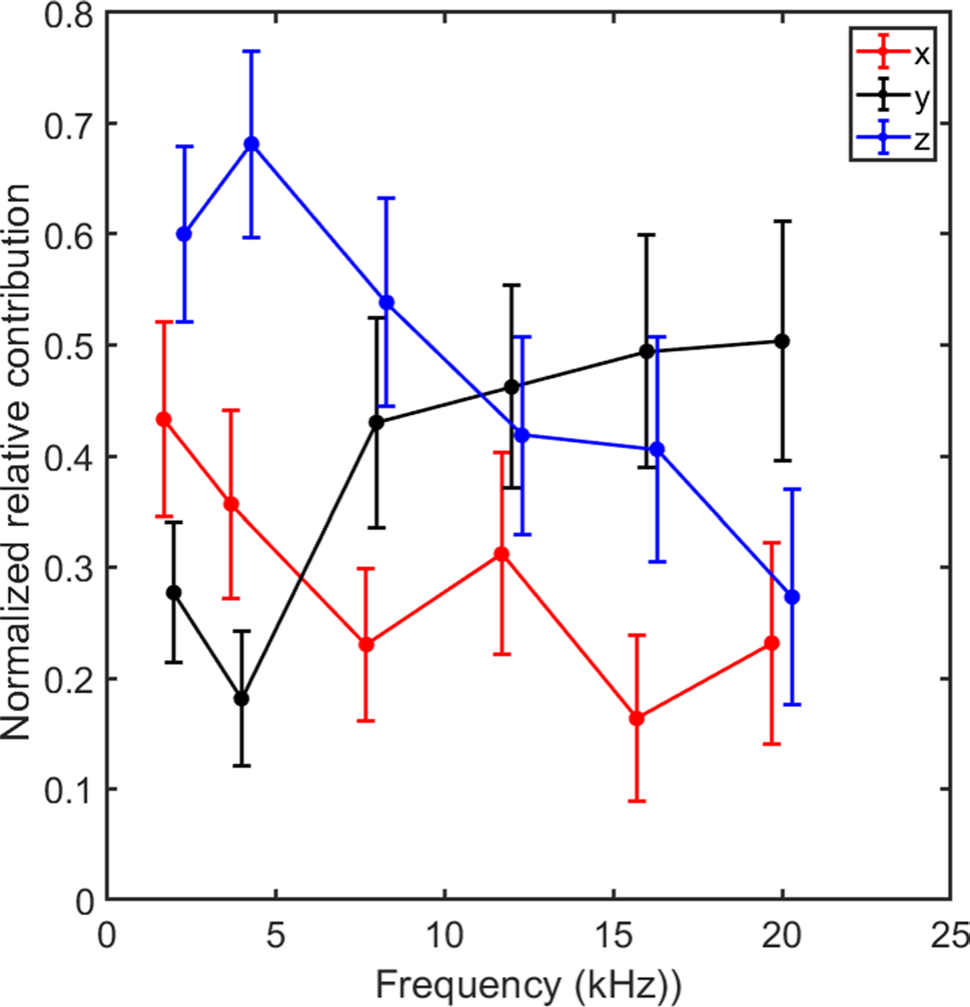
The normalized relative contributions on the hearing excitation from the x, y, and z direction BC vibrations at the cochlear promontory in guinea pigs.

The results of this analysis are presented in Fig. 6. The relative contribution in the z direction (*S*_*z*_) dominates at frequencies between 2 and 8 kHz with values between 0.55 and 0.68 where the maximum relative contribution from the other two direction falls between 0.4 and 0.5. This suggests that at these frequencies, the vibration in the z direction influence the hearing perception more than vibrations in the other two directions. This can also be seen in the variance and absolute threshold error estimates in Figs. 4 and 5 where the estimation based on the velocity magnitude in the z direction alone gave lower error values than those based on the x and y directions alone. At the higher frequencies, 12 to 20 kHz, the maximum values are found in the y direction, but it is only at the highest frequency of 20 kHz that the value in the y direction is clearly greater than that in the z direction. The least importance has the vibration in the x direction and the lowest values are found in this direction for frequencies of 8 kHz and above. When these results are interpreted in the cochlear anatomy presented in Fig. 1B, the vibration in-line with the normal direction of the stapes footplate (***d***_***1***_) is most sensitive at frequencies between 2 and 8 kHz, while the direction in-line of the base to apex (***d***_***3***_) of the cochlea is most important at the highest frequencies. The least important direction of the three vectors presented in Fig. 1B is the direction between the oval and round windows (***d***_***2***_). The results in Fig. 6 can be interpreted as the most important direction for a hearing perception by BC in the guinea pig is vibration in the z direction for frequencies at and below 8 kHz while the vibration in y direction is more important at frequencies between 12 and 20 kHz.

## Discussion

This study aimed to investigate the influence from different vibration directions on the perception of BC sound. This was accomplished by providing stimulation in 5 different directions at the vertex of a guinea pig head using a cubic brass connector. The rationale for using different stimulation directions at the guinea pig vertex was to achieve different combinations of the orthogonal vibrations at the cochlea that could be used to estimate their influence producing a sound perception by BC. To achieve this goal, the combinations of orthogonal vibrations at the cochlea should be as different as possible for the 5 stimulation attachments. Optimally, the vibrations at the cochlea should be one-dimensional with vibrations solely in the x, y and z directions sequentially with the change of the stimulation direction. However, the vibration of the cochlear promontory here (cf. Fig. 2) indicate that a stimulation of the guinea pig head result in a vibration response at the cochlear promontory in all spatial dimensions. Similar findings have been reported in human heads where stimulation at the skull surface result in vibration in all three space dimensions at the bone encapsulating the inner ear^5,22,31^.

Not only were there vibration in all 3 spatial dimensions of the cochlear promontory with stimulation in different directions at the vertex, the resulting 3D velocities at threshold were correlated for the different stimulation directions (Fig. 3). This makes it difficult to disentangle the contribution from the different vibration directions. This was especially problematic at the highest frequencies where the correlations between the vibration directions at threshold exceeded 0.85 (16 and 20 kHz). The small differences at the highest frequencies in the nine methods’ ability to predict the threshold (Figs. 4, 5) is attributed to this high correlation. One reason for the high correlation was that two directions were excited pairwise in opposition, the front and back direction and the left and right direction. Theoretically, these excitations should result in equal velocity magnitude responses at the cochlea as stimulation in opposite directions would be the same as inverting the phase of the stimulation. However, in reality, the excitation directions were not completely aligned with the coordinate system, the cuboid used for attachment of the transducer was not placed exactly symmetrical on the vertex of the guinea pig skull, and the attachment positions on the cuboid were spatially separated by 6 mm, all leading to slightly different three dimensional response magnitudes at the cochlea even when the stimulations were applied in virtually the same directions. Even so, the responses in these stimulation pairs is expected to be more similar than stimulation in orthogonal directions. Also, as there is no stimulation pair for the top position, the outcome can be biased to the results obtained in the front–back and left–right directions, since these produced two response measurements while only one response measurement was obtained with stimulation at the top position. However, Fig. 2 did not reveal any apparent similarity in the vibration magnitudes at threshold between the front–back and left–right stimulation directions compared to the stimulation at the top position.

Among the nine different methods used to predict the hearing thresholds (Table 2), the MSE gave the best predictions. This is expected since the MSE method finds the combination of the velocities for x, y, and z directions that result in the smallest mean squared error between the threshold predictions. In the current study, the weights for the velocities were restricted to be positive. This was done since phase of the vibrations were omitted and including negative values as weights had a tendency to fit the parameters to the noise in the data. In the current measurement setup, the guinea pig CAP was used to estimate a threshold. In this process, time or phase data were lost and the thresholds were a function of the velocity magnitude in the three orthogonal direction. Therefore, the use of velocity phase became problematic as there were no phase measure of the CAPs. Omitting the phase and only using magnitude data of the velocities have most likely increased the variance of the estimated thresholds.

Dobrev and Sim^22^ suggested a method to compute the magnitude and phase for the skull vibration in the direction with the greatest motion. That type of computation could have been incorporated in the current study to characterize the three-dimensional spatial motion of the bone around the inner ear. However, the direction of the greatest vibration magnitude at the cochlea would be different between animals as well as between tested frequencies, and it would be difficult to draw conclusions relating to the directional sensitivity of the inner ear for BC excitation, which is the purpose of the current study. Since no direction dominated the magnitudes of the velocities in the three orthogonal directions (Fig. 2), the magnitude of the vibration in the direction of the greatest motion is similar to the RMS magnitude (Eq. 4.5), and the result using an analysis of the velocity magnitude in the direction of the greatest motion would be similar to the results obtained with RMS analysis conducted here.

There is general agreement among the parameters used for the MSE estimation. Figure 6 shows the overall contribution from each vibration direction for all guinea pigs tested. At the lower frequencies, at and below 8 kHz, the z direction seems to be most important while at the highest frequencies, at and above 16 kHz, the y direction was most important. In addition, the x direction was found overall least important as contributor to the hearing stimulation. Consequently, vibration in the different directions do have different impact for the excitation of the inner ear. These findings are corroborated in Fig. 5 where predicting the hearing thresholds by the z direction alone gave lower absolute threshold errors than the x and y directions at frequencies at 4 and 8 kHz. No general difference between the three directions could be found at the highest frequencies in Fig. 5. It should be noted that the sensitivity found for these specific directions depend on the specific anatomy of the guinea pig head and cannot be generalized to an arbitrary species. However, the results indicate that there are vibration directions that are more important than others for excitation of the inner ear by BC.

When these findings are interpreted in the anatomy of the inner ear (Fig. 1B, Table 1), the normal direction of the stapes footplate (***d***_***1***_), that is mainly in the z direction, gives the greatest contribution at frequencies up to 8 kHz while the direction from the base to the apex of the cochlea (***d***_***3***_), that is mainly in the y direction, is more important at the highest frequencies. Since the ***d***_***1***_ direction is in-line with the in-and-out motion of the stapes, and also in line with the middle ear ossicles vibration direction (TM to stapes), this can be an indication that the inertial effects of the middle ear ossicles^27,32^ influences the BC hearing in guinea pigs at these frequencies. This conclusion has support in the findings in our previous study on BC hearing in guinea pigs where gluing the stapes footplate to the surrounding bone depressed the BC sensitivity significantly at 2 and 4 kHz, but not so much at the higher frequencies^30^. In the human, studies have identified ossicular inertia to be less important than other contributors to BC hearing^28,29^.

The high-frequency influence from the ***d***_***3***_ direction can be explained by a compressional mode of the cochlear shell. In the guinea pig, the cochlea protrudes into the middle ear space with only a thin bony shell surrounding it. With vibration in the direction of the cochlear base to apex, due to the minute thickness of the surrounding bone, this whole structure can be compressed forcing a fluid flow towards the oval and round windows and thereby creating a sound pressure difference between the scala vestibuli and scala tympani that generates a hearing sensation. It should be noted that this type of excitation is different from the compressional mode believed to be present in the human where the cochlea is surrounded by thick and dense skull bone^18^.

One interesting finding was that the velocity in one direction predicted the thresholds better or equally well than adding all three velocities linearly or quadratically (Figs. 4, 5). This indicates that only using one dimensional vibration data are as good as using three-dimensional data for estimation of the hearing stimulation with BC sound. However, the one-dimensional direction that gave the best estimation varied with frequency, and some one-dimensional directions gave significantly worse estimates of the thresholds than adding all three directions. Consequently, using the vibration in all three directions avoids larger errors in the threshold estimation compared to using solely one-dimensional vibrations, unless the important vibration direction for stimulating the inner ear is known.

To our knowledge, this is the first study that experimentally investigates the inner ears’ sensitivity to vibrations in different directions with BC sound. In a study on human middle ear ossicles motion in temporal bone specimens, Stenfelt et al.^27^ showed that the relative stapes vibration, i.e. the in and out motion of the stapes in relation to the surrounding bone, was within 5 dB independently of the stimulation direction. The difference between the velocity magnitude of the bone in line with the stimulation and orthogonal to the stimulation direction was reported as 15 to 20 dB, meaning that the motion of the ossicles could not be explained by the out of plane motion of the bone itself. Consequently, even if the vibration of the bone around the middle ear in humans is orthogonal to the in and out motion of the stapes, relative motions appear in the in and out motion direction of the stapes. This motion is only slightly lower than motions obtained when the stimulation of the bone coincides with the in and out motion of the stapes. The result from the Stenfelt et al.^27^ study implies that motions in one direction of the bone can be transformed to another direction of the movable parts in the auditory system.

Kim et al.^26^ investigated the importance of the vibration direction of the inner ear during BC stimulation in a finite element model of the human inner ear. They reported that a vibration in line with the hook region of the cochlea resulted in greater basilar membrane velocities than stimulation in other directions. The direction of the hook region coincides with the direction between the oval and round windows in the human inner ear, and this direction was assumed most important in the modelling studies of Stenfelt^15,18,33^ as well. The result in the current study did not indicate that the direction between the oval and round window (***d***_***2***_) was more important than other directions. When investigating the variability measure and mean absolute error measure in Figs. 4 and 5, the ***d***_***2***_ direction gave results that were overall slightly worse than the results in the ***d***_***1***_ direction, but overall better than the ***d***_***3***_ direction. Consequently, the direction that is most sensitive for BC stimulation can differ between the guinea pig and the human.

One shortcoming in the study of Kim et al.^26^ was that only the inertial response of the cochlea was investigated, and that the model lacked the cochlear and vestibular aqueducts that has been found important for BC sound generation in the inner ear^18,33^. Even so, that study identified that there are directions of the BC vibration at the inner ear that is more efficient than others for hearing sound. That was also found in this study, especially at the lower frequencies there were directions that gave greater excitation of the inner ear compared to other directions. The current study did not try to identify the mechanisms for the difference in sensitivity with stimulation direction. It has here been speculated that a vibration direction in line with the direction between the oval and round windows would provide a greater inertial response in the cochlea, or that the compression component can be more excited when the stimulation is in line with the base to apex of the cochlea, other contributors to the inner ear sound pressure, such as sound radiated into the ear canal^34^, can be more efficient with other stimulation directions.

## Conclusion

Similar to findings in vibration experiments in humans, a stimulation in a specific direction on the surface of the guinea pig head result in response vibrations at the guinea pig cochlea in all three space dimensions. The best estimate of the hearing stimulation is obtained with a weighted sum of the vibration amplitudes in the three dimensions. However, only using one-dimensional response can be equally good as using a linear or quadratic summation of the three-dimensional velocities, but it can also result in much worse estimates. The results indicate that there are vibration directions at the cochlea, here in-line with the vibration direction of the stapes at lower frequencies and in-line with the base to apex of the cochlea at higher frequencies, which have an overall greater influence on the guinea pig BC response than other directions.

## Material and methods

This study was approved by the regional ethics board in Linköping and all experiments followed EU Directive 2010/63/EU for animal experiments.

### Guinea pigs

The experiments were conducted in 22 male guinea pigs (Dunkin–Hartley). The guinea pigs had a mean weight of 478 g and were between 6 and 10 weeks old at the time of the experiment. These animals were also included in a previous study, and a more detailed information of the preparation is provided there^30^.

The guinea pig was anaesthetized with an intraperitoneal injection of Xylazine (10 mg/kg) and Ketamine (40 mg/kg). Additional injections of Xylazine and Ketamine was given at approximately intervals of 40 min to maintain deep anaesthesia. Oculentum simplex APL was provided to prevent the guinea pigs’ eyes from drying while bupivacaine (0.2 mg/kg) was administered to the guinea pigs’ ear canal. The Preyer's reflex was present in the guinea pigs and after the injections the fur on the vertex and around the ear was removed. A heating pad with a temperature of 37 °C was used to keep the guinea pig warm.

The surgery involved an opening into the bulla which required an incision from the vertex down to the back of the ear. The soft tissue was removed and the bulla was opened to expose the middle ear structures and the round window (RW). The activity of the inner ear was obtained by electrophysiology measurements and one silver electrode was placed on the RW while two needle electrodes were placed in the homolateral back leg and the ipsilateral cheek.

### Measurement set-up

A computer connected I/O board with 1 output channel and 4 input channels (National Instruments USB-4431) was used for signal generation and recording. An amplifier (NPI, EXT-02F-1) amplified the RW electrode signal 10,000 times which was then sampled at a frequency of 102.4 kHz by the I/O board. The output channel of the I/O board provided the signal to a BC transducer, which was a motor unit from a BAHA Classic bone conduction hearing aid (Cochlear Inc.) that was attached to the guinea pig vertex. This transducer has a size of 15 × 15 × 8 mm and a weight of 7 g. The vibrations of the guinea pig’s inner ear that was generated by the BC transducer was measured by a three-dimensional Laser Doppler Vibrometer (LDV), which consisted of a Polytech CLV-3D sensor head and CLV-3000 controller. The three laser beams from the LDV was focused on the same spot on the guinea pig’s cochlear promontory, between the oval window (OW) and RW.

To minimize the electrical noise during the electrophysiological measurements, the guinea pig was positioned inside an electrically and acoustically shielded chamber. A specially written software in LabView was used to generate the stimulus and record the electrophysiology signals. A brass cuboid adaptor was glued to the guinea pig bony vertex with dental cement (3 M ESPE Durelon carboxylate cement) (Fig. 1A). This 6 × 6 × 10 mm adaptor had threaded holes at the centre of all surfaces and the bone conduction vibrator could be rigidly attached in 5 directions on this adaptor (four at the sides and one at the top). This setup enabled vibrations to be applied in three orthogonal directions.

### Measurement procedure

The hearing measurements were conducted with the guinea pig positioned inside the shielded chamber. The stimulation had a total length of 10 ms consisting of a 6 ms long tone burst with 1 ms sine windowed rise and fall and 2 ms silence. This gave 100 stimulations per second that were present with alternating polarity for 4 s, resulting in a total of 400 stimulation presentations for each level and frequency. The measurements started at 2 kHz with a BC level of around 40 dB above threshold (40 dB HL_BC_guinea_pig_). After finalizing the stimulation and recording at 2 kHz, the test continued sequentially at the test-frequencies (2, 4, 8, 12, 16, and 20 kHz). The estimation of 40 dB HL_BC_guinea_pig_ was based on the results of BC thresholds in guinea pigs in a previous study^30^. After the measurements at all frequencies were conducted at a stimulation level, the level was decreased by 10 dB and the measurements were repeated with the decreased level at all test frequencies starting at 2 kHz. The level reduction continued down to 60 dB below the initial level, meaning that 7 stimulation levels for each frequency were measured. Based on the signal from the RW electrode, the compound action potential (CAP) was obtained for each stimulation level and frequency by averaging the 400 responses for each frequency and level. The BC measurements were repeated for all five stimulation directions (top, left, right, front, back). Figure 1A illustrates a guinea pig with the adapter for the stimulation attached to the bony vertex and also shows the coordinate system used for the current study where x-direction is ventral, y-direction is posterior and z-direction is left.

After finalizing the CAP measurements, the animal was euthanized by an injection of 1 ml pentobarbital sodium (60 mg/ml) in the abdomen. The vibration measurement of the cochlear promontory was conducted immediately after the euthanasia by placing the guinea pig on a vibration isolation table and aiming the three laser beams on the same spot on the cochlear bone between the OW and RW. A 2 s long chirp signal with frequencies from 1 to 50 kHz was supplied to the BC transducer and the resulting vibration on the cochlear bone were measured by the LDV and recorded by the I/O card. The Matlab function *tfestimate* was used to estimate the velocity based on the stimulation signal. The measurements were averaged 20 times and the velocities at the corresponding frequency bins in the estimate function were extracted. The bin size was 50 Hz and the signal to noise ratio for the extracted frequencies were between 15 and 40 dB depending on frequency and direction. The stimulation from all 5 positions on the adapter were obtained by changing attachment of the BC transducer to the top, right, left, front and back positions. In this way, the 3D vibration at the cochlea was obtained for all five different BC transducer attachments.

### Analysis

**Figure 7:**
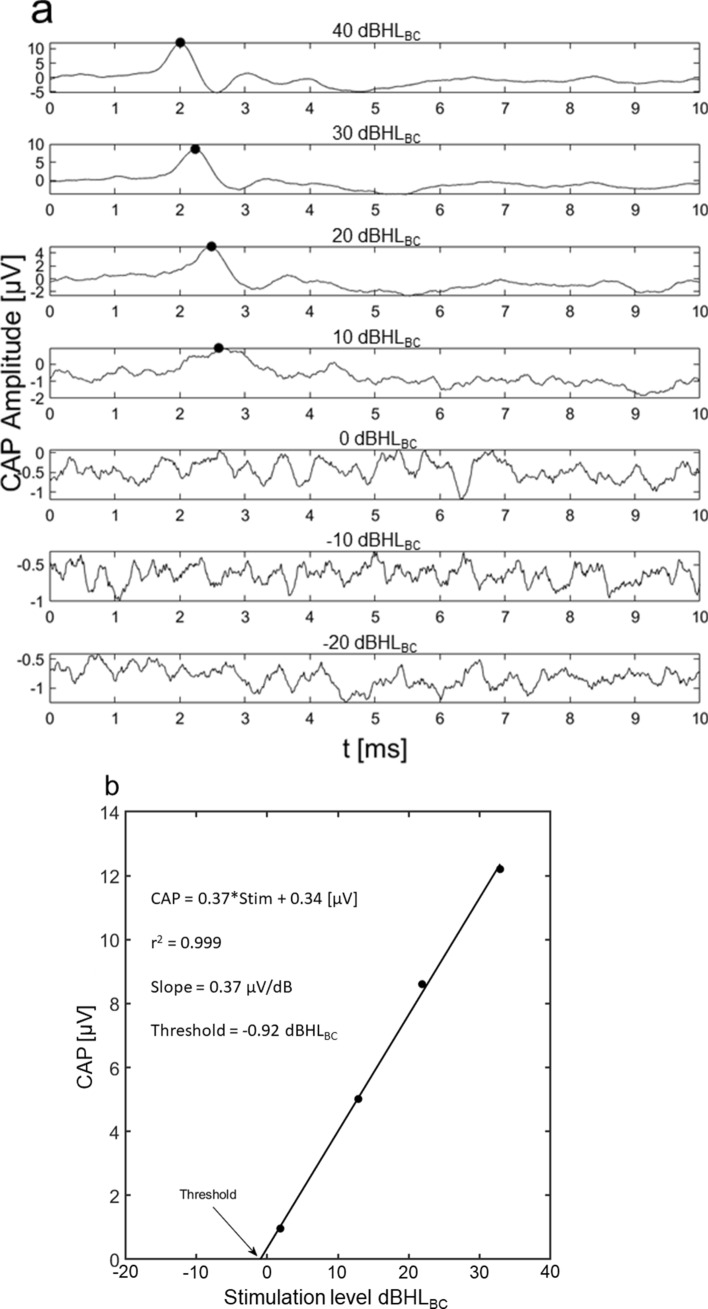
CAP measurements in the guinea pig. (**A**) A typical result of CAP measurement with BC stimulation. The peak voltage used for the analysis is indicated with a dot on the amplitude traces. (**B**) An example of the regression calculation used to obtain the threshold levels. The current example is for a stimulation frequency of 8 kHz.

All analysis was done using Matlab. The magnitude of the P1 wave in the CAP was identified manually by first averaging the 400 measured responses with alternating polarities. The averaging of alternating polarities reduces the contribution from cochlear microphonic potentials in the measurements. Figure 7a shows a typical result of the CAP identification where the CAP wave P1 from the 4 strongest stimulation levels were identified while the results at the three weakest stimulation levels had no identifiable CAPs. The hearing threshold level was defined as the stimulation level that produced a CAP wave P1 of 0 V. This was achieved by fitting the CAP wave P1 potentials by a linear slope in relation to the stimulation level in dBs, using the Matlab function *polyfit*. Figure 7b illustrates the linear fitting of the CAP potentials and the resulting estimated threshold level. This method was used to obtain the hearing threshold for all five directions of BC stimulation at all frequencies.

**Figure 8:**
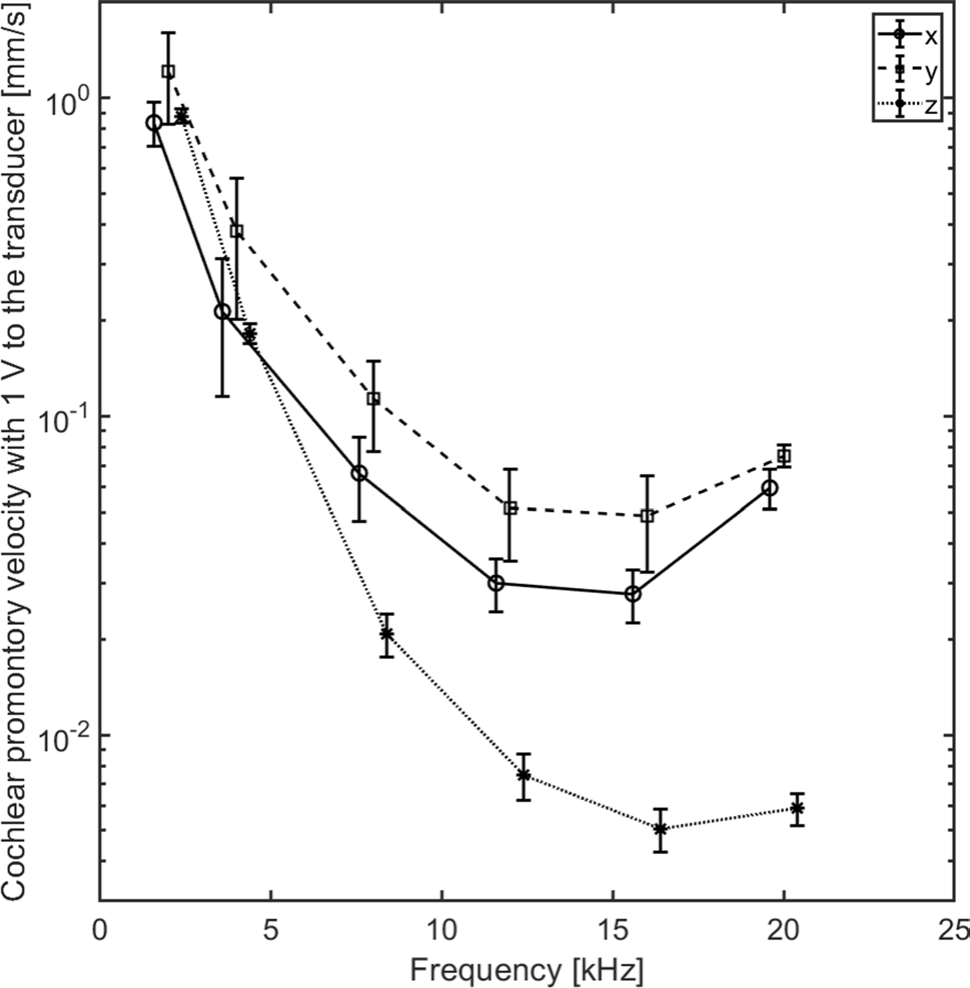
The average cochlear promontory velocity in three orthogonal directions when the stimulation is at the top of the guinea pig vertex. The stimulation is 1 V to the BC transducer. The error bars indicates the standard error of the mean.

The stimulation voltage that resulted in 0 V CAP was computed (here termed threshold voltage) for each frequency and stimulation direction. Then the relationship between the voltage to the BC transducer and the vibration velocity in the three orthogonal directions of the cochlear promontory was obtained using the 3D LDV (Fig. 8). Based on these computations, the vibration velocities at threshold in three orthogonal directions were calculated for each stimulation direction in each animal.

It is previously not known which vibration direction or combination of vibration directions at the cochlea that are responsible for a BC perception, and nine different combinations were analysed here. Three of those combinations were the velocity magnitudes in the x, y, and z directions alone, one was the linear sum of the vibration magnitudes in the three directions, one was the RMS (root mean square) of the 3D velocity magnitude (Table 2). The sixth velocity function was a linear combination of the three orthogonal velocity magnitudes where the coefficients *a*, *b*, and *c* were determined by a restricted minimum mean square error method, hereafter referred to as the MSE method (Table 2). The last three functions were based on the cochlear anatomy and the three vectors identified in Fig. 1B with ***d***_***1***_ being the vector in the direction normal to the stapes footplate, ***d***_***2***_ being the vector from the oval to round window, and ***d***_***3***_ being the direction of the centre of the cochlea from the base to the apex (Table 2).

By comparing how well these different combinations predict the hearing thresholds with stimulation in different directions, and thereby different combinations of 3D cochlear velocity magnitudes, the relative importance of cochlear vibration in a certain direction can be analysed.
